# Effective microbial molecular diagnosis of periodontitis-related pathogen *Porphyromonas gingivalis* from salivary samples using *rgpA* gene

**DOI:** 10.5808/gi.22076

**Published:** 2023-03-31

**Authors:** Jinuk Jeong, Yunseok Oh, Junhyeon Jeon, Dong-Heon Baek, Dong Hee Kim, Kornsorn Srikulnath, Kyudong Han

**Affiliations:** 1 Department of Bioconvergence Engineering, Dankook University, Yongin 16890, Korea; 2 Department of Biological Sciences, Dankook University, Cheonan 31116, Korea; 3 Department of Oral Microbiology and Immunology, College of Dentistry, Dankook University, Cheonan 31116, Korea; 4 Department of Anesthesiology and Pain Management, Dankook University Hospital, Cheonan 31116, Korea; 5 Animal Genomics and Bioresource Research Unit (AGB Research Unit), Faculty of Science, Kasetsart University, Bangkok 10900, Thailand; 6 Center for Bio Medical Engineering Core Facility, Dankook University, Cheonan 31116, Korea; 7 Department of Microbiology, College of Science & Technology, Dankook University, Cheonan 31116, Korea; 8 HuNbiome Co., Ltd, R&D Center, Seoul 08503, Korea

**Keywords:** molecular diagnosis, *Porphyromonas gingivalis*, quantitative real-time PCR, *rgpA* gene

## Abstract

Importance of accurate molecular diagnosis and quantification of particular disease-related pathogenic microorganisms is highlighted as an introductory step to prevent and care for diseases. In this study, we designed a primer/probe set for quantitative real-time polymerase chain reaction (qRT-PCR) targeting *rgpA* gene, known as the specific virulence factor of periodontitis-related pathogenic bacteria ‘*Porphyromonas gingivalis*’, and evaluated its diagnostic efficiency by detecting and quantifying relative bacterial load of *P. gingivalis* within saliva samples collected from clinical subjects. As a result of qRT-PCR, we confirmed that relative bacterial load of *P. gingivalis* was detected and quantified within all samples of positive control and periodontitis groups. On the contrary, negative results were confirmed in both negative control and healthy groups. Additionally, as a result of comparison with next-generation sequencing (NGS)–based 16S metagenome profiling data, we confirmed relative bacterial load of *P. gingivalis*, which was not identified on bacterial classification table created through 16S microbiome analysis, in qRT-PCR results. It showed that an approach to quantifying specific microorganisms by applying qRT-PCR method could solve microbial misclassification issues at species level of an NGS-based 16S microbiome study. In this respect, we suggest that *P. gingivalis*–specific primer/probe set introduced in present study has efficient applicability in various oral healthcare industries, including periodontitis-related microbial molecular diagnosis field.

## Introduction

Periodontal disease is one of the most common oral diseases that occur with inflammation inside or outside the tissues surrounding the dental structure (e.g., gums, gingival, and periodontal site), and it has the potential to cause other oral diseases, such as tooth loss [1,2]. In particular, *Porphyromonas gingivalis* is a gram-negative anaerobic microorganism that inhabits the subgingival plaque or saliva, and is classified as a component of periodontitis-associated core pathogenic bacteria (red complex) that affects host immune response and causes chronic periodontitis disease. Therefore, many oral healthcare-related industries have focused on an accurate diagnosis of *P. gingivalis* and research to inhibit intraoral colonization [3].

The quantitative real-time polymerase chain reaction (qRT-PCR) method is generally used in the direct to customers–based molecular diagnostic fields as a powerful tool for quickly and accurately diagnosing infected or not of certain pathogenic microorganisms [4,5]. Although the next-generation sequencing (NGS)–based metagenome sequencing method (e.g., 16S microbiome or shotgun metagenome sequencing methods) is possible to determine various microbial communities within each body site associated with human diseases, the qRT-PCR method is still preferred for researchers to detect and quantify specific microorganisms due to the time-cost burden of experiments and analysis of NGS technology [6,7]. Moreover, the high fluorescence sensitivity of qRT-PCR equipment is optimized for specific gene detection and quantification, making it more efficient to manufacture and commercialize a qRT-PCR–based microbial diagnostic kit that detects particular pathogens [8,9]. Although various molecular diagnostic kits or analytic platforms have been launched based on qRT-PCR technology, the market for microbial molecular diagnosis related to periodontal disease is still small compared to other similar fields such as the probiotic industry based on the gut microbiome study. One of the various reasons related to it is that the clinical sampling method performed to diagnose periodontal disease can cause discomfort to consumers in an invasive way that generally requires scratching microbial plaque within the subgingival site and is so difficult to detect in a healthy oral environment [10-12]. However, several recently conducted NGS-based oral microbiome studies have shown that microbial composition detected from saliva or mouthwash can somewhat represent the microbial community in the subgingival plaque sample [10,12]. In particular, Kim et al. [12] showed through the comprehensive 16S microbiome study that there is a significant difference in the relative bacterial frequency of *P. gingivalis* within saliva samples between periodontitis patients and healthy subjects.

In this study, we designed a specific primer and probe sets for detecting *P. gingivalis* within saliva samples collected from periodontitis patient (four adult per each group) and healthy groups through qRT-PCR method. The specific primer and probe sets were designed to target the coding region of the bacterial virulence factor '*rgpA*' gene (or arg-gingipain A), which has been founded to have a high-expression rate during the biofilm formation process of *P. gingivalis* [13]. We showed that the difference in relative bacterial load of *P. gingivalis* between each comparison group, which was detected and quantified through the qRT-PCR method, is similar to the NGS-based 16S V3–V4 metagenome sequencing results performed using the same clinical sample. Moreover, we cross-validated the species-specificity of the primer and probe sets through comparative experiments using positive control (two strains belonging to *P. gingivalis* species) and negative control (genomic DNA pools including other bacterial strains). Therefore, through this application report, we demonstrate that the *P. gingivalis*–specific primer and probe sets designed in this study have been confirmed high species-specificity that may be applied to the microbial molecular diagnosis of periodontal disease. 

## Methods and Results

### *P. gingivalis*–specific primer and probe sets design method

First, we designed a species-specific primer and probe sets for detecting and quantifying the bacterial load of *P. gingivalis* within human saliva samples through qRT-PCR, and the process was as follows.

#### Target gene selection

The *rgpA* is a *P. gingivalis*–specific gene, one of the various virulence gene complexes of *P. gingivalis*, known to increase the risk of periodontal disease by inhibiting the host immune defense system and contributing to the initial inflammatory response and host oral tissue damage around the gums [14].

#### Obtain of coding sequence information

We obtained the sequence information (FASTA format) of the *rgpA* gene coding sequence region (CDS) for 22 different *P. gingivalis* strains (at the strain level) on the National Center for Biotechnology Information (NCBI DB) reference database (Supplementary Table 1).

#### Selection of target-specific sequence regions based multiple sequence alignment method

**Fig. 1.**
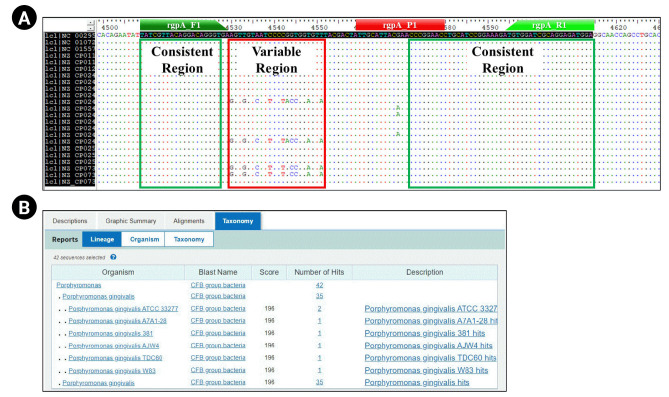
Primer/probe set design process for detection and quantification of relative bacterial load for *Porphyromonas gingivalis*. These figures show the design process of the primer/probe set for quantitative real-time polymerase chain reaction to detect and quantify the relative bacterial load of *Porphyromonas gingivalis*. (A) Multiple sequence alignment results using the Bioedit 7.2 ver. bioinformatics tool show that the primer/probe was designed within consistent regions (highlighted by green box) on the *rgpA* gene of all *P. gingivalis* strains (strain level) annotated on the NCBI database (rgpA_F1: forward primer sequence region; rgpA_R1: reverse primer sequence region; rgpA_P1: probe sequence region). (B) Basic Local Alignment Search Tool (BLAST) test results showing the species-specificity for *P. gingivalis* of primer set.

For the selection of primer sequence that can detect all 22 different *P. gingivalis* strains, we performed the multiple sequence alignment (MSA) of each CDS information using the BioEdit 7.2v software (Fig. 1A). We selected two consistent regions confirmed through the MSA method as forward and reverse primer sequences (Table 1).

#### *In silico* test

We pre-validated the suitability of the experimental application (Tm value, GC%, and potential for primer-dimer to form, etc.) about the selected primer pair using the ‘Oligo calc (http://biotools.nubic.northwetern.edu/)’ and ‘Oligo Analysis (http://www.operon.com/tools/oligo-analtsis-tool.aspx)’ open web tool. Next, we confirmed the *P. gingivalis*–specificity about each primer sequence and amplification region contained in each primer pair through the NCBI Nucleotide BLAST (https://blast.ncbi.nlm.nih.gov) (Fig. 1B).

#### Specific-probe design

Finally, we designed a specific probe in the region between each selected primer sequence (Table 1, Fig. 1A). The fluorescent reporter dye and quencher applied to the probe design are 6-FAM (6-carboxyfluorescein) and SFCQ1 (SFC probe, South Korea), which were attached to the 5' and 3' end regions on the selected probe sequence, respectively.

### Comprehensive validation for primer/probe-specificity using qRT-PCR method

We set up the comparison groups to verify experimental detection accuracy of the pre-designed *P. gingivalis*–specific primer/probe set (Table 2). A total of eight people (four periodontitis and four healthy oral groups) participated in this study, and we obtained saliva samples from them for identifying the bacterial load of *P. gingivalis* within oral cavity. All clinical experiments (recruitment of participants and collection of clinical samples) conducted for this study were approved by the Institutional Review Board of Dankook University Hospital (IRB numbers: 2020-10-015). All clinical examinations were performed by a dentist, who measured pocket depth, clinical attachment loss, gingival index, and plaque index through the full arch (Table 3). All collected clinical specimens were used to extract metagenomic DNA (mDNA) for polymerase chain reaction (PCR), qRT-PCR, and 16S microbiome analysis. The mDNA was extracted using the QIAamp PowerFecal Pro DNA Kit (QIAGEN, Hilden, Germany), and all experimental processes were performed according to the formal protocol guide provided in the Kit. The ‘ZymoBIOMICS standard DNA’, which belongs to the negative control group, is a mixed DNA sample in which the genomic DNAs of 10 different bacterial species (*Listeria monocytogenes, Pseudomonas aeruginosa, Bacillus subtilis, Saccharomyces cerevisiae, Escherichia coli, Salmonella enterica, Lactobacillus fermentum, Enterococcus faecalis, Cryptococcus neoformans*, and *Staphylococcus aureus*) are at a uniform rate (Zymo Research, Irvine, CA, USA). The experimental validation process for primer/probe activity is as follows.

#### PCR validation for confirming primer specificity

**Fig. 2.**
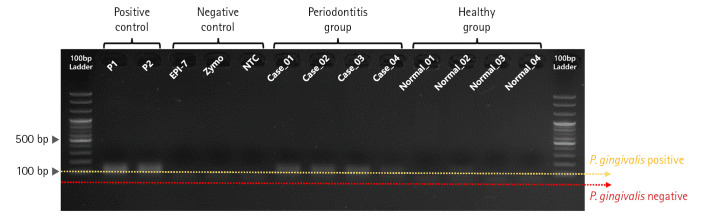
Polymerase chain reaction (PCR) validation result for evaluating primer binding activity of *Porphyromonas gingivalis*–specific primer set. Gel-electrophoresis showing the results of evaluating primer binding activity for the *rgpA* gene region on the *P. gingivalis* genome through PCR validation.

First, we performed PCR validation to confirm whether the pre-designed primer binds specifically to the *rgpA* gene CDS region on the *P. gingivalis* genome (Fig. 2). As a result of PCR validation, we confirmed that a correct target amplicon DNA band (approximately 110 bp) was located within samples corresponding to positive control and periodontitis groups. In contrast, amplicon DNA was not identified within samples corresponding to the negative control and healthy group.

#### Comparison of bacterial load about *P. gingivalis* within each sample using qRT-PCR

**Fig. 3.**
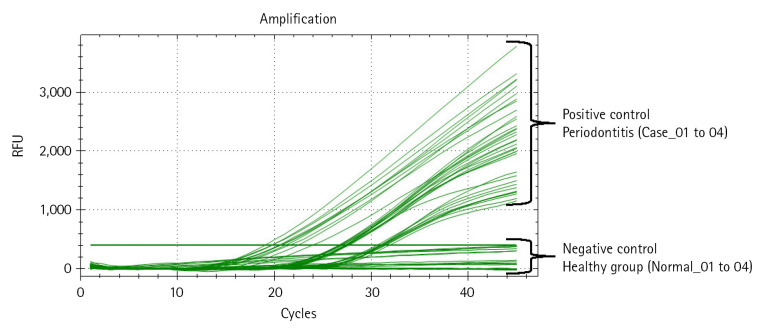
Quantitative real-time polymerase chain reaction (qRT-PCR) result reflecting the relative bacterial load of *Porphyromonas gingivalis* within each DNA sample. The amplification plot shows whether the relative bacterial load of *P. gingivalis* was detected within all samples in each comparison group. The x-axis on the plot indicates the running cycle number of qRT-PCR, and the y-axis indicates relative fluorescence units (RFU).

Next, we performed qRT-PCR to confirm the relative bacterial load of the *P. gingivalis* within each comparison group (Table 4, Fig. 3, Supplementary Table 2). Prior to the experiment, the concentration of all double-strand DNA samples was checked through the Qubit Fluorometer 4.0 v at the Center for Bio-medical Engineering Core Facility (Dankook University, South Korea) and 1× dsDNA HS Assay kit (Thermo Fisher Scientific, Waltham, MA, USA), and then normalized to the consistent concentration (10 ng/μL). This process is essential to compare the Ct value (cycle threshold) reflecting the relative frequency of *P. gingivalis* on the same mDNA concentration within saliva samples of each clinical subject (healthy and periodontitis). The same experimental conditions were set by normalizing the other microbial genomic DNA concentrations (positive and negative controls) equally with the clinical comparison group. After normalizing all DNA concentrations, to confirm the relative bacterial load of *P. gingivalis* as various DNA concentrations range, we performed qRT-PCR by diluting each template DNA (10^1^, 10^0^, 10^-1^, 10^-2^) through 10-fold serial dilution method (using CFX Opus 96 Real-Time PCR System, Bio-Rad, Hercules, CA, USA; The running condition of the qRT-PCR is as follows: pre-denaturation 95.0°C, 10 min; denaturation 95.0°C, 10 s; annealing 64.0°C, 15 s; elongation/flour detection 72.0°C, 15 s; and total PCR cycle was 45). As a result of qRT-PCR, we confirmed through the Ct value measurement results that the *P. gingivalis* *rgpA* gene was detected from all diluted DNA samples within the positive control and periodontitis groups. In contrast, *P. gingivalis* detection through confirming *rgpA* gene amplification could not be confirmed within the healthy and negative control groups (denoted ‘N/A’ in Table 4 and Supplementary Table 2). Although high-average Ct values (31.01_10 ng; 33.31_1 ng; 36.98_0.1 ng; 39.21_0.01 ng) were calculated from one sample in the periodontitis group (Case_04), our data show that *P. gingivalis* with low relative abundance within saliva sample can also be detected via qRT-PCR.

#### Comparison of bacterial detection between qRT-PCR and NGS-metagenome profiling

Finally, we checked the relative bacterial frequency of *P. gingivalis* within all samples of the healthy and periodontitis groups (four samples per each group) through the NGS-based 16S metagenome profiling analysis using the QIIEM2 microbiome analysis pipeline and compared it with the qRT-PCR result (Supplementary Table 3). The 16S microbiome analysis about eight clinical samples was performed through bacterial 16S V3-V4 amplicon sequencing using pre-extracted mDNA samples (Table 4), and the Illumina Miseq sequencer platform (300PE sequencing, Illumina, San Diego, CA, USA) was applied to the generation of raw sequencing data. We analyzed the paired-end 16S V3-V4 raw sequencing read files (i.e., fastq.gz files) using two bioinformatics pipelines: QIIME2 and DADA2 (version 1.2.0). The executing software for QIIME2 was Python software and for DADA2 was R (version 3.3.2). First, the sequencing reads were sorted using unique barcodes for each PCR product. The barcode, linker, and primer sequences were then removed from the original sequencing reads. And the removed reads were merged paired-end reads using FLASH v 1.2.11. The merged reads containing two or more ambiguous nucleotides, those with a low-quality score (average score < 20), or reads shorter than 300 bp, were filtered out. All pre-filtered sequence data quality control and feature table construction were completed using the Divisive Amplicon Denoising Algorithm 2 (DADA2) in the QIIME 2 plugin, which detects and corrects amplicon errors and filters out PhiX chimeric sequences. The pre-processed reads from each sample were used to calculate the number of Amplicon Sequence Variants (ASVs). The number of ASVs was determined by clustering the sequences from each sample using a 99% sequence identity cut-off using DADA2 pipeline-based QIIME2 software. Taxonomic relative abundance was counted with Naïve Bayes classifier using a confidence threshold of 0.7% derived from the ASVs data for each sample. The classified microbial composition based on SILVA v138 16S rRNA gene database was normalized using the value calculated from the taxonomy abundance count divided by the number of pre-processed reads for each sample. The relative bacterial frequency of *P. gingivalis* in each group identified through microbiome analysis was as follows: Healthy group (Normal_01: 0.00, Normal_02: 0.00, Normal_03: 0.00, and Normal_04: 0.00); Periodontitis group (Case_01: 0.00, Case_02: 0.16, Case_03: 0.16, and Case_04: 0.00). As with the qRT-PCR results, we confirmed that *P. gingivalis* was not detected within all samples of the healthy group. However, unlike the qRT-PCR results, we identified that the relative bacterial frequency of *P. gingivalis* was not calculated within two samples (Case_01 and Case_04) of the periodontitis group. These results demonstrated that qRT-PCR also sensitively captured the low abundance for *P. gingivalis*, which was not confirmed by the NGS-based metagenome sequencing method.

## Discussion

We introduced a high-sensitivity primer/probe set that can specifically detect and quantify the relative bacterial load of periodontitis-related pathogenic bacteria ‘*Porphyromonas gingivalis*’ within human saliva sample. This primer/probe set was designed to target the *P. gingivalis*–specific gene, *rgpA* gene, and has never been applied as a target gene to diagnose the *P. gingivalis*. Moreover, we suggest that the microbial detection accuracy of our primer/probe set is excellent because we designed a primer/probe set to cover the *rgpA* gene of all *P. gingivalis* strains (strain level) annotated on the NCBI reference database. By comparing bacterial detection and quantification results for *P. gingivalis* between NGS-based metagenome profiling data and qRT-PCR, we cross-validated that the qRT-PCR method using the *rgpA* gene-specific primer/probe set showed higher detection sensitivity than the microbiome analysis targeting the bacterial 16S ribosomal RNA gene. This result shows that microbial misclassification problem at the species level due to high sequence similarity within the hyper-variable regions on the bacterial 16S rRNA gene occurring from 16S microbiome analysis can be overcome through the qRT-PCR method [15,16]. In this respect, we suggest that the *P. gingivalis*–specific primer/probe set introduced in the present study has efficient applicability in various oral healthcare industries, including the periodontitis-related microbial molecular diagnosis field.

## Figures and Tables

**Table 1.** Sequence information of *Porphyromonas gingivalis*–specific primer and probe sets

Primer/Probe name	Sequence (5' to 3')	Length (mer)
*rgpA*_Forward	TATCGTTACAGGACAGGGTG	20
*rgpA*_Reverse	TCCATCTCCTGCGATCCACA	20
*rgpA*_Probe	56-FAM/TACGAACCCGGAACCTGCATCC/3SFCQ1	22

**Table 2.** Sample information applied to this study

Groups	Sample name	DNA type	Extracted raw DNA concentration (ng/μL)
Positive control	*Porphyromonas gingivalis* ATCC 49417	Single species DNA	13.50
	*P. gingivalis* ATCC 33277		4.00
Periodontitis	Case_01	Metagenomic DNA	46.00
	Case_02		47.20
	Case_03		26.60
	Case_04		15.30
Healthy	Normal_01	Metagenomic DNA	47.20
	Normal_02		33.20
	Normal_03		29.80
	Normal_04		21.20
Negative control	*Epidermidibacterium keratini* EPI-7^T^	Single species DNA	10.90
	ZymoBIOMICS standard DNA	Multiple species DNA	10.00
	NTC (DW)	None DNA	-

**Table 3.** Demographic characteristic and clinical parameters of periodontal disease group and healthy group

Characteristic	Healthy	Periodontitis	p-value
Sex			
Male	3	4	
Female	1	0	
Age (y) (mean ± SD)	28.1 ± 10.55	38.8 ± 13.40	<0.01
Examination			
PD (mm)	2.49 ± 0.51	5.97 ± 2.27	<0.01
CAL (mm)	2.31 ± 0.22	6.12 ± 2.09	<0.01
GI	0.17 ± 0.46	1.69 ± 0.94	<0.01
PI	0.35 ± 0.62	1.28 ± 0.92	<0.01

SD, standard deviation; PD, probing depth; CAL, clinical attachment loss; GI, gingival index; PI, plaque index

**Table 4.** Comparison of bacterial load about *Porphyromonas gingivalis* using qRT-PCR

Groups	Samples	Ct value of each dilutied DNA sample							
		10 ng (raw concentration)		1 ng (10^-1^ dilution)		0.1 ng (10^-2^ dilution)		0.01 ng (10^-3^ dilution)	
		Avg	SD	Avg	SD	Avg	SD	Avg	SD
Positive control	*P. gingivalis* ATCC 49417	19.67	0.04	22.34	0.18	25.12	0.04	27.85	0.04
	*P. gingivalis* ATCC 33277	19.45	0.06	22.02	0.25	24.8	0.01	27.33	0.02
Periodontitis	Case_01	27.28	0.09	30.33	0.06	33.45	0.11	37.04	0.01
	Case_02	27.49	0.02	30.18	0.19	33.13	0.03	35.51	0.12
	Case_03	26.65	0.04	30.02	0.15	32.57	0.10	35.01	0.19
	Case_04	31.01	0.18	33.31	0.05	36.98	0.79	39.21	0.08
Healthy	Normal_01	N/A	N/A	N/A	N/A	N/A	N/A	N/A	N/A
	Normal_02	N/A	N/A	N/A	N/A	N/A	N/A	N/A	N/A
	Normal_03	N/A	N/A	N/A	N/A	N/A	N/A	N/A	N/A
	Normal_04	N/A	N/A	N/A	N/A	N/A	N/A	N/A	N/A
Negative control	*Epidermidibacterium keratini* EPI-7^T^	N/A	N/A	N/A	N/A	N/A	N/A	N/A	N/A
	ZymoBIOMICS standards	N/A	N/A	N/A	N/A	N/A	N/A	N/A	N/A
	NTC (D.W.)	N/A	N/A	N/A	N/A	N/A	N/A	N/A	N/A

qRT-PCR, quantitative real-time polymerase chain reaction; Avg, average Ct value for each triplicate calculations; SD, standard deviation value for each triplicate calculations; N/A, no detection.

## Supplementary Materials

Supplementary Table 1. NCBI database annotation information about *rgpA* gene of each *Porphyromonas gingivalis* strains

Supplementary Table 2. Supplementary Table 2. Results of qRT-PCR quantification for comparing bacterial load about *Porphyromonas gingivalis* of each sample

Supplementary Table 3. Bacterial relative abundance data at the species level of all samples within healthy and periodontitis groups
